# Is it possible to manipulate perfectionism in children?

**DOI:** 10.1186/2050-2974-3-S1-O27

**Published:** 2015-11-23

**Authors:** Tracey Wade, Kate Fairweather-Schmidt

**Affiliations:** 1 School of Psychology, Flinders University, Australia

## 

Reducing unhelpful perfectionism has been shown to produce transdiagnostic outcomes in adults, reducing depression, anxiety and weight concerns. There is limited evaluation of the impact of reducing unhelpful perfectionism in adolescents but no evaluation in children. The aim of the current pilot study was to investigate whether perfectionism could be manipulated in children aged 10-12 years. Classes of school students were randomly assigned to one of two conditions: perfectionism (N=57) and assessment only-control (N=68). Students in the active condition participated in 2 lessons. Assessment occurred on three occasions: baseline, post-intervention, and 2-week follow-up. Relative to control, the perfectionism condition was found to decrease unhelpful perfectionism, suggesting that children benefited from the intervention.

